# Mucilage from Yellow Pitahaya (*Selenicereus megalanthus*) Fruit Peel: Extraction, Proximal Analysis, and Molecular Characterization

**DOI:** 10.3390/molecules28020786

**Published:** 2023-01-12

**Authors:** María Carolina Otálora, Andrea Wilches-Torres, Jovanny A. Gómez Castaño

**Affiliations:** 1 Grupo de Investigación en Ciencias Básicas (NÚCLEO), Facultad de Ciencias e Ingeniería, Universidad de Boyacá, Tunja 150003, Colombia; 2 Grupo Química-Física Molecular y Modelamiento Computacional (QUIMOL®), Escuela de Ciencias Químicas, Universidad Pedagógica y Tecnológica de Colombia, Tunja 150003, Colombia

**Keywords:** mucilage, hydrocolloid, pitahaya, polysaccharide, biopolymer, by-product

## Abstract

Mucilage is a hydrophilic biopolymeric material of interest in the food industry due to its high content of dietary fiber, antioxidant activity, and gelling and thickening capacities, which is present in high concentration in agricultural by-products, such as the peel of cacti fruits. In this work, the powdered mucilage extracted from the peel of yellow pitahaya (*Selenicereus megalanthus*) fruit was characterized using a multi-technical approach that included proximal analysis (proteins, lipids, crude fiber, ash, and carbohydrates), as well as structural (FTIR, NMR, UPLC-QTOF-MS, and X-ray diffraction), colorimetric (CIEL*ab* parameters), morphological (SEM), and thermal (DSC/TGA) methods. Likewise, its total content of dietary fiber and polyphenols, as well as its antioxidant activity, were determined. This dried mucilage presented a light pale yellow-reddish color, attributed to the presence of betalains (bioactive pigments with high antioxidant activity). The FTIR spectrum revealed functional groups associated with a low presence of proteins (5.45 ± 0.04%) and a high concentration of oligosaccharides (55.26 ± 0.10%). A zeta potential of −29.90 ± 0.90 mV was determined, denoting an anionic nature that favors the use of this mucilage as a stable colloidal dispersion. UPLC-QTOF-MS analysis revealed a major oligosaccharide composition based on galacturonic acid units in anionic form. SEM micrographs revealed a cracked morphology composed of amorphous and irregular particles. According to the DSC/TGA results, this mucilage can be introduced as a new source of hydrocolloids in food processes since it has high thermal stability that has been manifested up to 373.87 °C. In addition, this biopolymer exhibited a high content of polyphenols (25.00 ± 0.01-g gallic acid equivalent (GAE)/100-g sample), dietary fiber (70.51%), and antioxidant activity (1.57 ± 0.01 mmol Trolox equivalents/kg of sample). It was concluded that this mucilaginous material presents sufficient physicochemical and functional conditions to be used as a nutritional ingredient, thus giving valorization to this agricultural by-product.

## 1. Introduction

In response to new consumer trends, the food industry has turned its attention to the use of green polymers based on oligosaccharides as a replacement for conventional hydrocolloids, such as maltodextrin, alginate, gum arabic, and carrageenan, among others, which offer limited nutritional value to the final product. Because of this search, the mucilage contained inside the fruits and leaves of some plant species, mainly from the Cactaceae family, has emerged as an attractive natural hydrocolloid alternative due to its sustainability, biodegradability, low costs, and, most of all, its desirable functional, nutritional, and physicochemical properties [1,2].

The yellow pitaya (*Selenicereus megalanthus*) is an exotic subtropical plant belonging to the Cactaceae family, whose fruit of yellow skin and white pulp has desirable organoleptic properties (delicate aroma and flavor), phytochemical content (essential fatty acids and ascorbic acid), and antioxidant activity (phenolic compounds). The world and Colombian production of pitahaya for 2018 was 2,100,000 t and 13,250 t, respectively [3]. The massive consumption of this fruit leads to the generation and significant disposal of by-products (epicarp and part of the mesocarp), which represent approximately one-third of the gross weight of the fruit and are destined for animal consumption or are simply left to decompose outdoors [4]. However, this by-product is a rich store of essential nutrients, including lipids, proteins, carbohydrates, and bioactive substances (antioxidants), such as betalains (betacyanins and betaxanthins; the latter used as natural colorants in the food industry), as well as pectin and dietary fiber [5]. This exfoliation also contains collenchyma and parenchyma tissues with mucilaginous cells [6].

Mucilage is a water-soluble biopolymer rich in oligosaccharides of an anionic polyelectrolytic nature due to the presence of carboxyl and hydroxyl groups [7], which exhibited a non-Newtonian shear-thinning flow behavior [8]. The mucilage polysaccharide is composed mainly of monosaccharide units such as L-arabinose, D-xylose, L-rhamnose, D-galactose, mannose, glucose, arabinose, and uronic acids (D-galacturonic acid) in various proportions [9]. This natural hydrocolloid is a rich source of dietary fiber with a powerful antioxidant action due to its high content of polyphenolic compounds [1,10]. This hydrocolloid presents a high water-holding capacity, solubility, and appropriate emulsifying and foaming properties [8].

The food industry shows an increasing interest in the incorporation of mucilage in its formulations due to its biocompatibility, biodegradability, non-toxicity, and good cost-benefit ratio, as well as its thickening, gelling, and viscosifying properties and ability to modify texture and stabilize [1]. As far as we know, there are no reports on the physicochemical and nutritional characterization of the powdered mucilage extracted from the peel of the yellow pitahaya fruit. Consequently, this work aimed to extract the mucilage from the peel of the yellow pitahaya fruit, evaluate its proximal composition and characterize it structurally, morphologically, and thermally, as well as determine its content of polyphenols, dietary fiber, and antioxidant activity. The results of our research show outstanding structural and functional conditions of this mucilage, thus giving value to the discarded peels of yellow pitahaya fruits as a natural source of hydrocolloids with healthy properties for the design of new food products.

## 2. Results and Discussion

### 2.1. Proximal Chemical Analysis

As shown in Table 1, a relatively low protein content (5.45%) was found in the dry mucilage extracted from the peel of the yellow pitahaya fruit, which is close to the protein content of 5.85% reported for the pericarp of the fruit of red pitaya (*Hylocereus* sp.) [11]. The protein content in the pitaya fruit peel mucilage is lower than that reported for other natural hydrocolloids such as *Cordia myxa* mucilage (8.90 ± 0.16%) [12], guar gum (8.19%) [13], and *Eruca sativa* seed mucilage (9.75%) [14]. Furthermore, a low protein content in mucilage samples may be associated with protein loss during the ethanol purification process (Section 2.3) [14], which makes pitaya fruit peel mucilage present a limited emulsifying power [14]. In contrast, the proximal analysis revealed the predominance (55.3%) of carbohydrates (oligosaccharides) in the mucilage of the pitaya fruit peel, higher than that reported in other natural hydrocolloids such as gum arabic (49.74%) [15] but lower than the value (81.4%) reported for the pericarp of the red pitaya fruit [11] and *Eruca sativa* seed mucilage (67.97%) [14]. The high content of oligosaccharides in the mucilage of the yellow pitaya fruit peel makes this biopolymeric material have hydrophilic characteristics, which makes it a potential thickening agent with high gelling capacity, which could be suitable for developing new products in the food and pharmaceutical industries [12,16,17].

As expected for mucilage, the lipid content of yellow pitaya fruit peel mucilage was very low (0.90%), with a percentage close to that reported for locust bean gum (0.99%) [12] but higher than that reported for the pericarp of red pitaya fruit (<0.53%) [16]. This is consistent with the fact that the oily fraction is retained in the cell structure and not in the gel during the mucilage extraction process [18]. In contrast, high content of lipids in the mucilage can influence the stabilization of aqueous emulsions due to the formation of networks that reduce the movement of oil droplets, as well as the collision and coalescence of the latter [19].

An ash content of 12.6% was found in the yellow pitahaya fruit peel mucilage, which is very close to that reported for the pericarp of the red pitaya fruit (12.34%) [11] and higher than that reported for other mucilages, such as chia seed mucilage (10.1 ± 0.02%) [20] and *Eruca sativa* seed mucilage (10%) [14], but lower than that reported for commercial carrageenan gum (15.0%) [21]. This percentage of ash suggests a high content of minerals (calcium, magnesium, phosphorus, potassium, and others) that, on the one hand, can affect the technological and physicochemical properties of the polysaccharide biomaterial. On the other hand, it would give additional nutritional value to future food preparations based on this mucilage [19,22,23].

The yellow pitaya fruit peel mucilage presented a higher content of dietary fiber (70.51 g/100 g) compared to that found for the red pitaya fruit pericarp (60.1 g/100 g) [11] and for the mucilage of chia seeds (51.26 g/100 g) [24], which makes the yellow pitaya fruit peel mucilage a potential gelling agent with functional properties since its intake is associated with prebiotic effects and the production of short-chain fatty acids [7]. 

### 2.2. Antioxidant Capacity and Color Parameters

As shown in Table 2, the total content of phenolic compounds (TPC) in the mucilage extracted from the peels of the pitaya fruit (25.0-g GAE/100 g of sample) is higher than that reported only for the shell of the pitaya fruit (15.94 ± 0.93 mg GAE/g of sample) [7]. Thus demonstrating that these substances accumulate preferentially in the mucilaginous fraction of the peel. This content of polyphenols gives a significant antioxidant capacity (1.57 TEAC) to the pitaya fruit peel mucilage, which is higher than that reported for other similar natural hydrocolloids [25]. Such favorable antioxidant capacity is mainly attributed to the presence of betalains and other tyrosine-derived pigments present in the fruit peel [7], which can donate their electrons and sequester the ABTS cation radical. This result makes pitaya fruit peel mucilage an attractive antiradical biomaterial for the design of new antioxidant products in the food industry.

The color parameters listed in Table 2 show how the mucilage from pitaya fruit peels has a low luminosity value (47.9), tending to a whitish-brown color, possibly caused by pigments. The values of the parameters a* (0.39) and b* (10.06) indicate that the color of the mucilage tends toward red and yellow tones. These chromatic coordinates are attributed to the presence of betalains (betacyanins and betaxanthins), the most important pigments of the genus *Hylocereus*, co-eluted while extracting the mucilage from the peel, which are responsible for the red (betacyanins) and yellow (betaxanthins) tones [7]. Furthermore, the hue angle (h_ab_*) and low saturation (C_ab_*) characterize the color of this mucilage as a light pale reddish yellow.

### 2.3. Structural Characterization

#### 2.3.1. Fourier-Transform Infrared (FTIR) Spectroscopy

**Figure 1:**
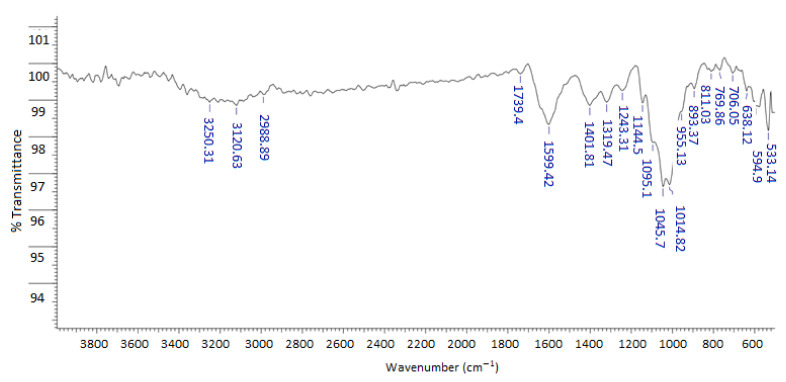
FTIR spectra of powdered mucilage extracted from yellow pitahaya fruit peel.

The FTIR spectrum of the powdered mucilage extracted from the peel of the yellow pitahaya fruit is shown in Figure 1. The broad band of medium intensity centered at 3250 cm^−1^ was attributed to the stretching of the hydroxyl groups (O-H), both from alcohol (R-OH) and carboxylic acid (-C(O)-OH) moieties, involved in the intramolecular hydrogen bond (HB) commonly found in polysaccharide structures [25]. The signals observed at 3120 and 2988 cm^−1^ were assigned to the asymmetric and symmetric C-H vibrations of the methylene (CH_2_) groups, respectively. The low-intensity absorption located at 1739 cm^−1^ was associated with the amide I band [26], which is consistent with the low percentage of protein found in the proximal analysis (Table 1). Consequently, the band of medium intensity at 1600 cm^−1^ was assigned to the carboxylic group, COOH, likely corresponding to the uronic acid (galacturonic acid types) fragments of the main oligosaccharide component [27]. The bands observed at 1401 and 1329 cm^−1^ were associated with C-O vibrations in the carboxylic groups. The signals at 1243 and 1144 cm^−1^ were assigned to the C-O-H and C-O-C bending modes, respectively. The intense band with peaks at 1045 and 1014 cm^−1^ was related to the presence of C-O-C bonds between the monomers that form the polymer structure [25,26].

#### 2.3.2. X-ray Diffraction (XRD) Analysis 

**Figure 2:**
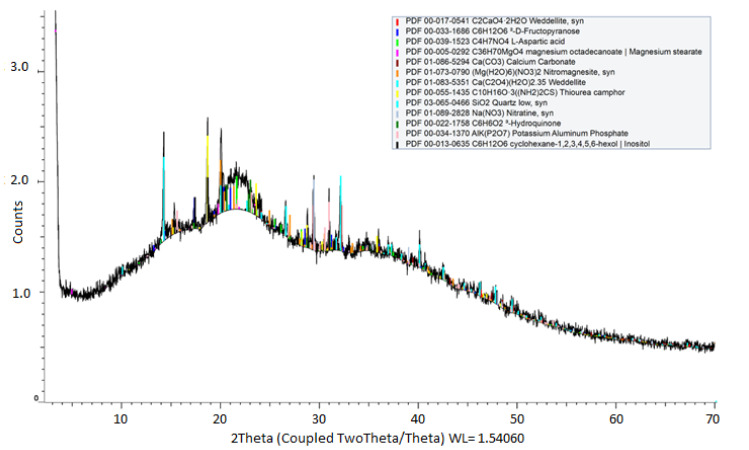
Powder diffraction pattern of a solid sample of mucilage extracted from pitaya fruit peel (black line). In color, the different crystallographic phases are identified.

The powder X-ray diffraction pattern of the solid sample of the mucilage extracted from the peels of the pitaya fruit (Figure 2) allowed the identification of thirteen different crystalline phases, distributed in (i) seven inorganic minerals: sodium nitrate (NaNO_3_), calcium carbonate (CaCO_3_), *syn*-nitromagnesite ((Mg(H_2_O)_6_)(NO_3_)_2_), weddellite (Ca(C_2_O_4_)(H_2_O)_2.35_), *syn*-weddellite (C_2_CaO_4_·2H_2_O), *syn*-quartz (SiO_2_), and potassium aluminum phosphate (AlK(P_2_O_7_)), and (ii) in six molecular crystals: D-fructopyranose (C_6_H_12_O_6_, MW = 180.2 g/mol), L-aspartic acid (C_4_H_7_NO_4_, MW = 133.1 g/mol), magnesium stearate (C_36_H_70_MgO_4_, MW = 591.2 g/mol), camphor thiourea (C_10_H_16_O_3_((NH_2_)_2_CS), MW = 260.35 g/mol), hydroquinone (C_6_H_6_O_2_, MW = 110.1 g/mol), and cyclohexane-1,2,3,4,5,6-hexol inositol (C_6_H_12_O_6_, MW = 180.2 g/mol). Such various crystalline phases agree with the relatively high percentage of ash (12.6%) determined in the proximal analysis (Table 1), from which a mineral composition based mainly on sodium, potassium, calcium, magnesium, silicon, and aluminum can be deduced.

#### 2.3.3. UPLC-QTOF-MS

**Figure 3:**
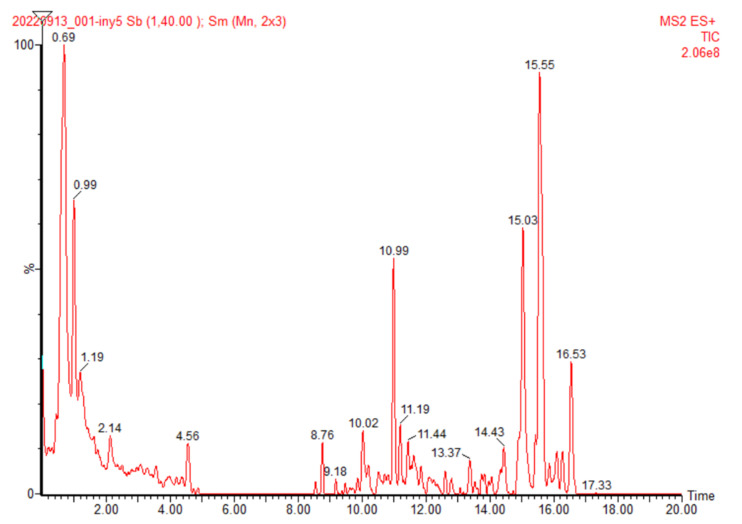
Peak chromatogram for an aqueous solution of mucilage extracted from yellow pitahaya fruit peels obtained by UPLC-QTOF-MS/MS.

The full ultra-high-performance liquid chromatogram (UPLC) for the aqueous solution of the mucilage extracted from pitaya fruit peels is presented in Figure 3. The overall profile of this chromatogram was very similar to the UPLC chromatogram we recently reported for the mucilage powder extracted from *Opuntia ficus-indica* (OFI) fruit peels [8], where both chromatograms are characterized by three predominant signals located at retention times (r.t) of 0.6–0.7, 11, and 16 min. These three signals are also observed in the chromatograms of the pitaya peel mucilage sample dissolved in water:acetonitrile (50:50) and water:methanol (50:50), see Figures S1 and S2 in the supporting information, but with a much simpler profile (i.e., fewer peeks) than that presented in the sample dissolved in water. The rest of the chromatographic peaks were related to compositional contributions below 10% and were left unassigned, except for the signal at r.t of 8.8 min whose *m/z* ratio of 111.4 Da [M + H]^+^ coincided with the molecular weight of hydroquinone (C_6_H_6_O_2_) detected by X-ray diffraction of mucilage powder (Section 2.3.2).

**Figure 4:**
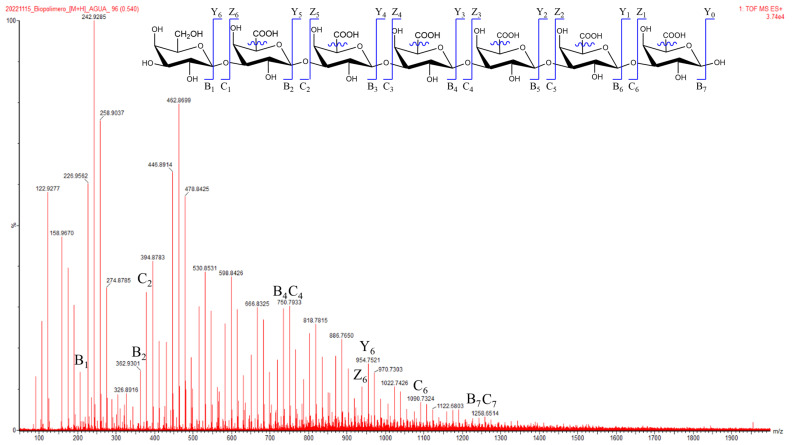
Positive ESI-MS spectrum of the major native oligosaccharide component (r.t of 0.7 min) of pitahaya fruit peel mucilage dissolved in water and measured at a collision offset voltage of 20 V. Fragmentation labels are indicated using the Domon nomenclature [24].

As in the case of the OFI fruit peel mucilage [8], the main peak at r.t of 15.6 min for pitahaya mucilage was related to an [M + H]^+^ *m/z* ratio equal to 290.5 Da (see ESI-MS spectrum in Figure S3 in supporting information) and could correspond to a catechin type metabolite. Likewise, the peak at r.t 11.0 min ([M + H]^+^ = 679.8 Da) was assigned to a hitherto undetermined betacyanin derivative, with an ESI-MS fragmentation pattern (Figure S4 in supporting information) at *m/z* ratios of 452.5 ([M + H]^+^ − 227), 343.4 ([M + H]^+^ − 336), 336.5 ([M + H]^+^ − 343), 226.4 (([M + H]^+^ − 452), 210.2 (base peak), and 100.2. The third peak, at r.t of 0.7 min, corresponded to the main oligosaccharide component (1874.4 Da) of the pitaya fruit peel mucilage, whose ESI mass spectrum in aqueous media is presented in Figure 4.

The mass fragments produced by ESI of the main oligosaccharide of the peel mucilage of the yellow pitaya fruit were obtained as molecular cations [M − H]^+^, [M + Na]^+^ and [M + nNa]^n+^ in the range of *m/z* 200 to 1300. As shown in Figure 4, the fragmentation pattern of the oligosaccharide presents equally spaced line separations of 15.97 *m/z*, attributable to losses of oxygen atoms from both hydroxyl groups and glycosidic bond breaks. The numerical analysis of the fragmentation sequence also reveals systematic losses of 67.99 *m/z*, which coincides with the *m/z* ratio for the [COOH + Na]^+^ cation, thus indicating uronic acid monosaccharide units. The fragmentation of the first unit of the nonreducing tail (i.e., B_1_ = 206.9 *m/z*) coincides with the monosaccharide unit of galactose [Gal + 2Na]^2+^, which with the presence of uronic acid units, allows us to elucidate a chain oligomeric formed by one galactose (Gal) and six galacturonic acid (GalA) units, whose mass coincides with the molecular cation C_7_ [Gal-[GalA]_6_-Na = 1258.7 *m/z*]. The other molecular cations can be derived by breaking the glycosidic bonds and carboxylic groups of the polysaccharide chain.

#### 2.3.4. Nuclear Magnetic Resonance (NMR)

1D-NMR spectra (^1^H, ^13^C, and DEPT/^13^C) and 2D-NMR spectra (COSY, HSQC, and HMBC) of a sample of yellow pitahaya fruit peel mucilage dissolved in DMSO-d6 are shown in Figures S4–S6 and S6–S9, respectively, in the Supplementary Information.

^1^H NMR (400 MHz, DMSO-d6), δ 5.34 (s, 1H), 4.87 (s, 2H), 4.45 (s, 9H), 3.52 (d, *J* = 7.0 Hz, 4H), 3.44–3.32 (m, 1H), 3.25–3.14 (m, 1H), 2.31 (s, 1H), 2.08 (t, *J* = 14.8 Hz, 1H), 1.29 (s, 4H), 1.27–1.17 (m, 1H), 1.12 (t, *J* = 7.0 Hz, 8H), 0.91 (dd, *J* = 6.9, 3.7 Hz, 1H), and 0.16–0.10 (m, 1H).

^13^C NMR (101 MHz, DMSO-d6), δ 75.70 (CH), 73.22 (CH), 73.10 (CH), 72.33 (CH), 56.61 (C), 56.51 (C), 40.59 (CH), 40.38 (CH), 40.16 (CH), 39.95 (CH), 39.75 (CH), 39.35 (CH), 29.3 (CH), and 1 18.99 (CH_3_).

### 2.4. Zeta Potential

The value of the zeta potential (ζ) of the powdered pitahaya fruit peel mucilage dispersed in water was −29.90 ± 0.90 mV, thus indicating an anionic nature [28,29,30,31,32]. This anionic response was mainly attributed to the presence of negatively charged carboxyl groups (COO^−^) in the galacturonic acid units, which is consistent with the results obtained by UPLC-QTOF-MS and FTIR measurements. Very similar negative zeta potentials were found in natural polymers such as alginate (−29.94 ± 1.45), xanthan gum (−30.67 ± 0.12), and pectin (−31.30 ± 0.14), as well as the synthetic polymer EUDRAGIT L100 (−29.88 ± 1.80) [33,34]. On the other hand, Otálora et al. [35] and Bouaouinea et al. [36] reported zeta potential values of −23.63 ± 0.55 and −23.00 mV for *Opuntia ficus-indica* fruit peel mucilage and *Opuntia ficus-indica* pad mucilage, respectively. According to Kang et al. [37], colloidal dispersions with absolute zeta potentials >30 were considered stable; meanwhile, dispersions with absolute zeta potentials <30 were considered unstable. This latter behavior was due to the weak repulsive forces that lead to a high aggregation tendency. Consequently, the ζ-potential ≈ 30 for pitahaya fruit peel mucilage will lead to the formation of stable colloidal dispersions, which can be attributed to its structure (presence of carbohydrates and proteins), molar mass, and predominantly negative charge [38].

### 2.5. Morphological Characterization

**Figure 5:**
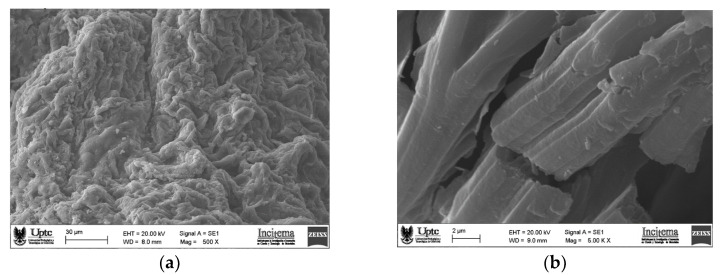
SEM micrograph images of the surface at 500× (**a**) and 5000× (**b**) of powder mucilage from yellow pitahaya fruit peel.

SEM micrographs of powdered pitaya fruit peel mucilage are presented in Figure 5. The surface structure of the mucilage captured at 500× magnification (Figure 5a) reveals a rough, cracked, and porous texture, accompanied by amorphous cavities with inequalities both in shape and size. This morphology could be associated with the low emulsifying capacity of the mucilage derived from its low protein content [14]. The image of the mucilage taken with a magnification of 5000× (Figure 5b) revealed small particles possibly corresponding to protein aggregates adhered to the carbohydrate blocks of the sample [11], which agrees with the results obtained by FTIR (Section 2.2). This microscopic morphology can affect the hydration capacity and other properties of the mucilage, such as viscosity [39].

### 2.6. Thermal Characterization

**Figure 6:**
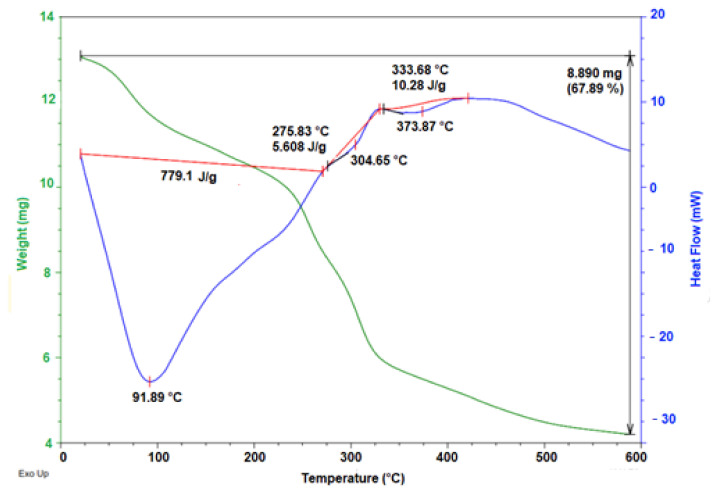
TGA/DSC thermogram of powder mucilage from yellow pitahaya fruit peel.

The thermogram of the powdered mucilage extracted from the peel of the yellow pitahaya fruit (Figure 6) revealed two main endothermic events. The first event occurred between 25 and 250 °C (peak 91.89 °C), with a related mass loss of 12.35%. This event was attributed to the loss of adsorbed and structural water, which is in accordance with the hydrophilic nature of the functional groups of the main polysaccharide, followed by a gelatinization process [40]. The second event occurred between 250 and 475 °C (peak 373.87 °C), with a mass loss of 67.89%. This event was attributed to the degradation of the polysaccharide backbone and subsequent decomposition/volatilization of the material. Similar thermal behavior was previously reported for mucilage extracted from the peel of *Opuntia dillenii* haw fruit (350 °C) [41] and gum arabic (322.7 °C) [42]. On their part, Otálora et al. [35] reported an increase in the thermal behavior of the mucilage extracted from the peel of the *Opuntia ficus-indica* fruit (423.10 °C).

According to the above, the mucilage extracted from the peel of the yellow pitahaya fruit is highly thermostable, which suggests that it can be used as a hydrocolloid in the design of new food products.

## 3. Materials and Methods

### 3.1. Chemicals and Reagents

Acetonitrile (41.05 g/mol, ≥99.9, CAS No 75-05-8) and formic acid (46.03 g/mol, >98%, CAS No 64-18-6) solvents (HPLC grade), as well as ethanol (46.07 g/mol, analytical grade, 97%, CAS No 64-17-5), were purchased from Merck (Darmstadt, Germany). DMSO-d6 (84.17 g/mol, 99.9 atom% D, CAS No 2206-27-1) was purchased from Cam-bridge Isotope Laboratories, Inc. (Tewksbury, MA, USA). 2,2′-azino-bis(3-ethylbenzothiazoline-6-sulfonic acid) (ABTS) was purchased from Sigma-Aldrich (St. Louis, MO, USA).

### 3.2. Plant Material

Fresh yellow pitahaya peels were collected from local restaurants in the city of Tunja, Boyacá, Colombia, washed with distilled water at room temperature, cut into small pieces, and processed immediately.

### 3.3. Mucilage Extraction

**Figure 7:**
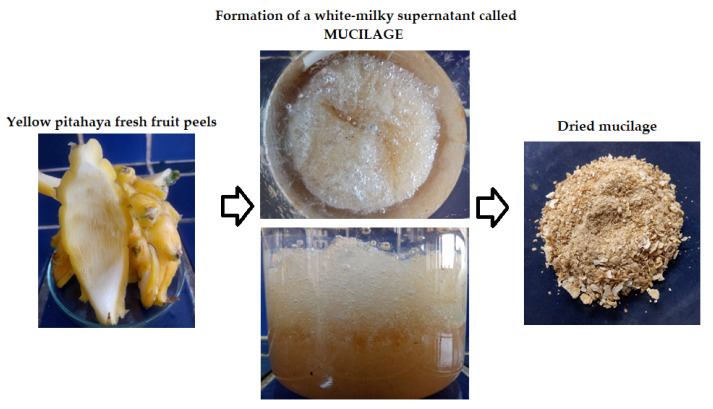
Selected photographs of the process of extracting mucilage from the peels of the fruit of yellow pitahaya.

The mucilage extraction was performed according to the methodology reported by Otálora et al. [35] with some modifications. The small pieces of pitahaya peels, without pulp parts, were placed in a 100 mL beaker to which distilled water was added at room temperature in a ratio of 1:2 *w/v* (peel:water) and left for 12 h. The hydrated peels were manually crushed to extract the gel. To the gel obtained, 95% ethanol was added in a ratio of 3:1 (ethanol:gel) to 18 °C, and the mixture was allowed to stand for 15 min without stirring until the formation of a milky-white supernatant corresponding to the mucilage from the peel of the pitahaya fruit. The mucilage was collected and then dried in an oven at 50 °C for 3 h. The dry material was macerated manually in a porcelain mortar and subsequently sieved through a 60 mesh until a fine powder was obtained (standard granulometry ≤ 250 μm). The powdered mucilage was placed in high-density polyethylene bags and stored in a desiccator at room temperature with a relative humidity of 30% until characterization. Selected photographs of the extraction and appearance of the mucilage powder from yellow pitahaya fruit peels are shown in Figure 7.

### 3.4. Proximal Chemical Analysis

The moisture, protein, lipids, crude fiber, and ash content of the powdered mucilage were determined according to the standard methods described by the Association of Official Analytical Chemists International (AOAC) [43]. Briefly, the moisture content was determined by employing the gravimetric approach (method 925.10), and protein concentration was determined by the Kjeldahl method using a correction factor of 6.25 (method 920.176). The lipid concentration was measured using Soxhlet extraction with petroleum ether (method 920.177). Crude fiber content was determined using acid hydrolysis followed by vacuum filtration (method 985.29), and ash content was determined by calcination at 550 °C in a muffle oven (method 900.02). The carbohydrate content (CC) was calculated as shown in Equation (1). The total dietary fiber content was determined using a total dietary fiber test kit (TDF-100A) provided by Sigma-Aldrich (St. Louis, MO, USA), which is based on the enzymatic–gravimetric method AOAC 985.29 [44].

(1) CC=100−(ash+fat+protein+crude fiber)

### 3.5. Total Phenolic Content

The total phenolic content was determined by the spectrophotometric method of Folin-Ciocalteu reported by Singleton and Rossi [45]. Around 500 mg of powdered mucilage was dissolved in 10 mL of methanol, stirred at room temperature, and centrifuged (3000 rpm, 15 min, 15 °C). 1 mL aliquot of the supernatant was mixed with 15 mL of water and 1 mL of 1 N Folin-Ciocalteu, and the mixtures were incubated for 2 h, and, after storage for 90 min at room temperature, the absorbance of the solution was read at 765 nm using a UV-vis spectrophotometer (V530, Jasco, Hachioji, Tokyo, Japan). A standard curve was constructed to quantify the total phenolic content using gallic acid at concentrations of 0 to 235 mg/L, and the results were expressed as g gallic acid equivalent (GAE)/100 g of sample on the dry weight.

### 3.6. Antioxidant Capacity

Trolox equivalent antioxidant capacity (TEAC) was measured using the method reported by Re et al. [46]. About 500 mg of mucilage powder was dissolved in 10 mL methanol, stirred at room temperature, and filtered through a Millipore membrane. The 0.5 µL liquid samples were mixed with 1 mL of ABTS solution, and their absorbance at 734 nm was read in a UV-vis spectrophotometer (V530, Jasco, Hachioji, Tokyo, Japan) using methanol as a blank. The results were expressed in mmol equivalents of Trolox/kg of sample (TEAC).

### 3.7. Color Parameters

The CIEL*ab* parameters (L*, a*, b*) of the powdered mucilage sample were measured using a colorimeter (CM-5, Konica Minolta Sensing, Inc., Osaka, Japan). The derived color parameters chroma (C*_ab_) and hue (*h_ab_*) were calculated using Equations (2) and (3), respectively.

(2) $$C_{ab}^{*}={\left[{\left(a^{*}\right)}^{2}+{\left(b^{*}\right)}^{2}\right]}^{1/2}$$

(3) $$h_{ab}=arctan\;\left[b^{*}/\;a^{*}\right]$$

### 3.8. Zeta Potential

The zeta potential of the powdered mucilage was measured using a NanoPlus TM 3 Particle Size Zeta Potential Analyzer (Norcross, GA, USA) at 25 °C and pH = 5.33. The powdered mucilage (602.8 mg) was dispersed in 100 mL of ultrapure deionized water using a magnetic stirrer (C-MAG HS 7 S000, IKA, Staufen im Breisgau, Germany) at 8000 rpm for 6 h at room temperature. The results were recorded as the average of triple replicates ± standard deviation.

### 3.9. Structural/Molecular Characterization

#### 3.9.1. UPLC-QTOF-MS

Full chromatographic separation (i.e., all dissolved components), the molecular weight analysis of the main biopolymeric component, including the average values (Mn, Mw, Mz, and Mz+1), and the polydispersity index (Mw/Mn) of the powdered mucilage were determined using an Acquity UPLC HClass-XEVO TQD (Waters Corporation, Milford, Massachusetts, USA). These UPLC-TQD analyses were performed in positive electrospray ionization (ESI) mode. Samples of 1 mg of the mucilage powder were dissolved in deionized water (solution 1), in water:methanol (50:50, solution 2), and in water:acetonitrile (50:50, solution 3). All samples were stirred for 6 h at room temperature and then centrifuged at 5000 rpm for 15 min, and the supernatant was filtered through a 0.45-micron Millipore filter. Aliquots (5 μL) were separated through an Acquity UPLC BEH C18 analytical column (2.1 mm × 100 mm, 1.7 µm particle size). The eluent system was composed of type I water and 0.1% formic acid (solvent system A), and acetonitrile and 0.1% formic acid (solvent system B) at a flow rate of 0.4 mL/min. The gradient elution program was set as follows: 0–5 min (95% A), 5–18 min (50% A), and 19–20 min (95% A). The following parameters were maintained: source temperature 120 °C, desolvation temperature 350 °C, desolvation gas flow rate at 800 L/h, and cone gas flow rate of 100 L/h. The cone and capillary voltages were set at 20 V and 2.5 kV, respectively. Some experiments were performed using the collision cell turned off to detect the molecular ions [M + H or M + Na].

#### 3.9.2. Fourier-Transform Infrared (FTIR) Spectroscopy

The FTIR spectrum of the powdered mucilage was measured on a Bruker Alpha ECO-ATR (Bruker, Karlsruhe, Germany) in the range of 4000–500 cm^−1^ with a resolution of 4.0 cm^−1^ and 24 cumulative scans using the attenuated total reflectance/reflection (ATR) technique.

#### 3.9.3. Nuclear Magnetic Resonance (NMR) Spectroscopy

The powdered mucilage structure was analyzed by one-dimensional (^1^H, ^13^C, and DEPT 135) and bi-dimensional (HSQC, HMBC, and COSY) NMR spectroscopy, using a Bruker Avance DPX 250 MHz spectrometer (Bruker, Karlsruhe, Germany) operating at 9.4 Tesla. Approximately 32 mg of the sample was solubilized in 0.6 mL DMSO-d6. All analyses were performed at 298 °C, and frequencies of 400.16 MHz and 100.63 MHz were used for ^1^H and ^13^C nuclei, respectively.

#### 3.9.4. X-ray Diffraction (XRD) Technique

X-ray diffraction analysis of powdered mucilage was performed on a Bruker D8 Advance DaVinci Geometry X-ray diffractometer (Bruker-AXS, Karlsruhe, Germany) with Lineal LynxEye detector using Cu-Kα radiation, produced at 40 kV and 40 mA. Data were recorded from the range 4° to 70° (step size of 0.02° and 0.6 s counting time for each step).

### 3.10. Morphological Characterization

The microscopic morphology of the powdered mucilage was evaluated by scanning electron microscopy (SEM) using an EVO MA 10-Carl Zeiss equipment (Oberkochen, Germany) operating at 20 kV. All samples were coated by gold–palladium sputtering before their examination.

### 3.11. Thermal Characterization

Thermogravimetric analysis (TGA)/differential scanning calorimetry (DSC) of powdered mucilage was performed on a TA Instrument (SDT Q600 V20.9 Build 20, New Castle, DE, USA). Argon was used as a purge gas (100 mL/min). The dried samples of powdered mucilage were placed in aluminum pans and heated from 20 to 600 °C at a heating rate of 10 °C/min.

### 3.12. Statistical Analysis

Proximal composition and color parameters, as well as total carotenoid content (TCC) and antioxidant capacity (TEAC), as presented in Table 1 and Table 2, respectively, were analyzed using analysis of variance (ANOVA). The date was reported as the mean ± standard deviation (*n* = 3).

## 4. Conclusions

The results of this work showed that mucilaginous material contained in fruit peels could be used as a functional ingredient with health-promoting properties. This biomaterial presented a faint red-dish-yellow color associated with the presence of betalains (betacyanins and betaxanthins), pigments with high antioxidant activity, which adhere to the main oligo-saccharide structure of the mucilage. The vibrational analysis by FTIR revealed the predominant presence of oligosaccharides and a low concentration of proteins, while the DRX studies of the powder allowed us to identify the crystalline structures that give minerals their solid sample. The microscopic morphology of the mucilage was described as a rough, cracked, and irregular structure. The DSC/TGA results revealed significant thermostability of the mucilage, with which this ingredient could be incorporated during formulation processes in the food industry. The solution behavior of the mucilage was approached using zeta potential, UPLC-QTOF-MS, and NMR spectroscopy. The mucilage behaved in an aqueous solution as an anionic dispersion, attributed to the formation of sodium acetate groups in the carboxylic terminals of galacturonic acid, the latter being the main monosaccharide of the biopolymer. Additionally, the proximal analysis of pitaya skin mucilage revealed a high accumulation of bioactive compounds (pigments and polyphenols), as well as a high amount of dietary fiber. Finally, the valorization of pitahaya peel in mucilage as a food fortification ingredient that can provide some additional health benefits is a promising alternative for the food industry that wishes to enter the “clean label” product market.

## 5. Patents

The results of this work are a structural part of the National Invention Patent Application No. NC2022/0007738 submitted for evaluation to the Superintendencia de Industria y Comercio of Colombia.

## Figures and Tables

**Table 1:** Proximal composition of mucilage extracted from yellow pitahaya fruit peel.

Component	Data (%)
Moisture	3.28 ± 0.17
Protein	5.45 ± 0.04
Lipids	0.90 ± 0.28
Ash	12.60 ± 0.14
Crude fiber	25.79 ± 0.28
Carbohydrates	55.26 ± 0.10
Total Dietary Fiber content	70.51

**Table 2:** Total phenolic content (TPC), antioxidant capacity (TEAC), and CIEL*ab* color space parameters for mucilage extracted from yellow pitahaya fruit peels.

TPC ^1^	TEAC ^2^	CIEL*ab* Color Space Parameters ^3^				
		L*	a*	b*	C_ab_*	h_ab_*
25.00 ± 0.01	1.57 ± 0.01	47.93 ± 0.05	0.39 ± 0.01	10.06 ± 0.02	10.07 ± 0.03	87.77 ± 0.06

^1^ Total phenolic content (TPC) is measured as g gallic acid equivalent (GAE)/100 g of sample in dry base. ^2^ Antioxidant capacity (TEAC) is measured as mmol Trolox equivalents/kg of sample in dry base. ^3^ CIEL*ab* Color Space Parameters.

## Data Availability

The data presented in this study are available on request from the corresponding author.

## Supplementary Materials

The following supporting information can be downloaded at: https://www.mdpi.com/article/10.3390/molecules28020786/s1, Figure S1: Peak chromatogram for a water:acetonitrile (50:50) solution of mucilage extracted from yellow pitahaya fruit peels obtained by UPLC-QTOF-MS/MS.; Figure S2: Peak chromatogram for a water:methanol (50:50) solution of mucilage extracted from yellow pitahaya fruit peels obtained by UPLC-QTOF-MS/MS.; Figure S3: Positive ESI-MS spectrum of the component at r.t of 15.6 min of pitahaya fruit peel mucilage dissolved in water and measured at a collision offset voltage of 20 V; Figure S4: ^1^H NMR spectrum (in DMSO-d6) of mucilage extracted from yellow pitahaya fruit peels; Figure S5: ^13^C NMR spectrum (in DMSO-d6) of mucilage extracted from yellow pitahaya fruit peels; Figure S6: DEPT/^13^C NMR spectrum (in DMSO-d6) of mucilage extracted from yellow pitahaya fruit peel; Figure S7: ^1^H-^1^H COSY NMR spectrum (in DMSO-d6) of mucilage extracted from yellow pitahaya fruit peels; Figure S8: HSQC 2D-NMR spectrum (in DMSO-d6) of mucilage extracted from yellow pitahaya fruit peels; Figure S9: HMBC 2D-NMR spectrum (in DMSO-d6) of mucilage extracted from yellow pitahaya fruit peels.
