# Supplementary material for: Mucilage from Yellow Pitahaya (Selenicereus megalanthus) Fruit Peel: Extraction, Proximal Analysis, and Molecular Characterization
Source: Molecules. 2023 Jan 12;28(2):786. doi: 10.3390/molecules28020786 (PMC9865909; doi:10.3390/molecules28020786)
Supplement: Supplementary file 1 [file molecules-28-00786-s001.zip › molecules-2141473-supplementary.pdf]

molecules

MDPI

Article

Supporting Information

Mucilage from Yellow Pitahaya (*Selenicereus megalanthus*) Fruit Peel: Extraction, Proximal Analysis, and Molecular Characterization

María Carolina Otálora [1,*], Andrea Wilches-Torres [1] and Jovanny A. Gómez Castaño [2,*]

[1] Grupo de Investigación en Ciencias Básicas (NÚCLEO), Facultad de Ciencias e Ingeniería, Universidad de Boyacá, Tunja, 150003, Colombia
[2] Grupo Química-Física Molecular y Modelamiento Computacional (QUIMOL®), Escuela de Ciencias Químicas, Universidad Pedagógica y Tecnológica de Colombia, Tunja, 150003, Colombia
* Correspondence: marotalora@uniboyaca.edu.co (M.C.O.); jovanny.gomez@uptc.edu.co (J.A.G.C.)

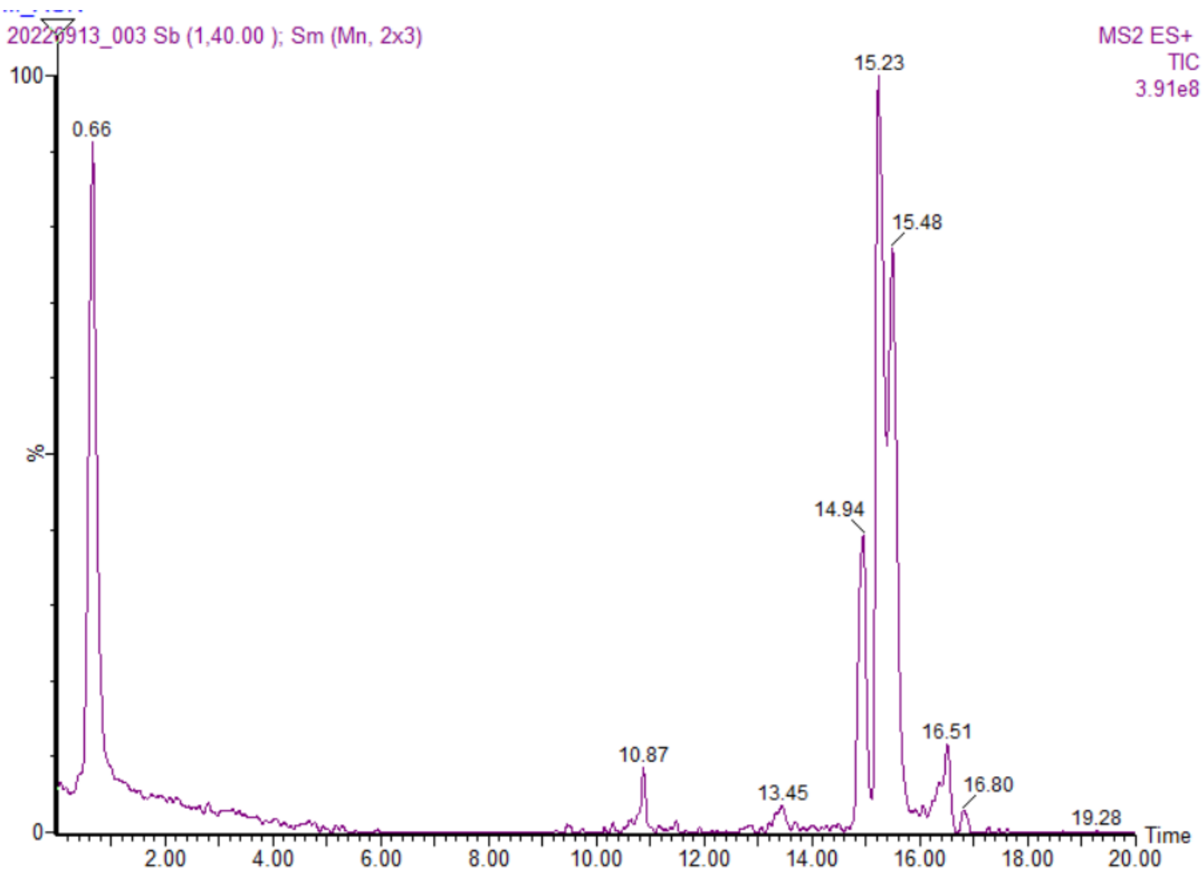

Figure S1. Peak chromatogram for a water:acetonitrile (50:50) solution of mucilage extracted from yellow pitahaya fruit peels obtained by UPLC-QTOF-MS/MS.

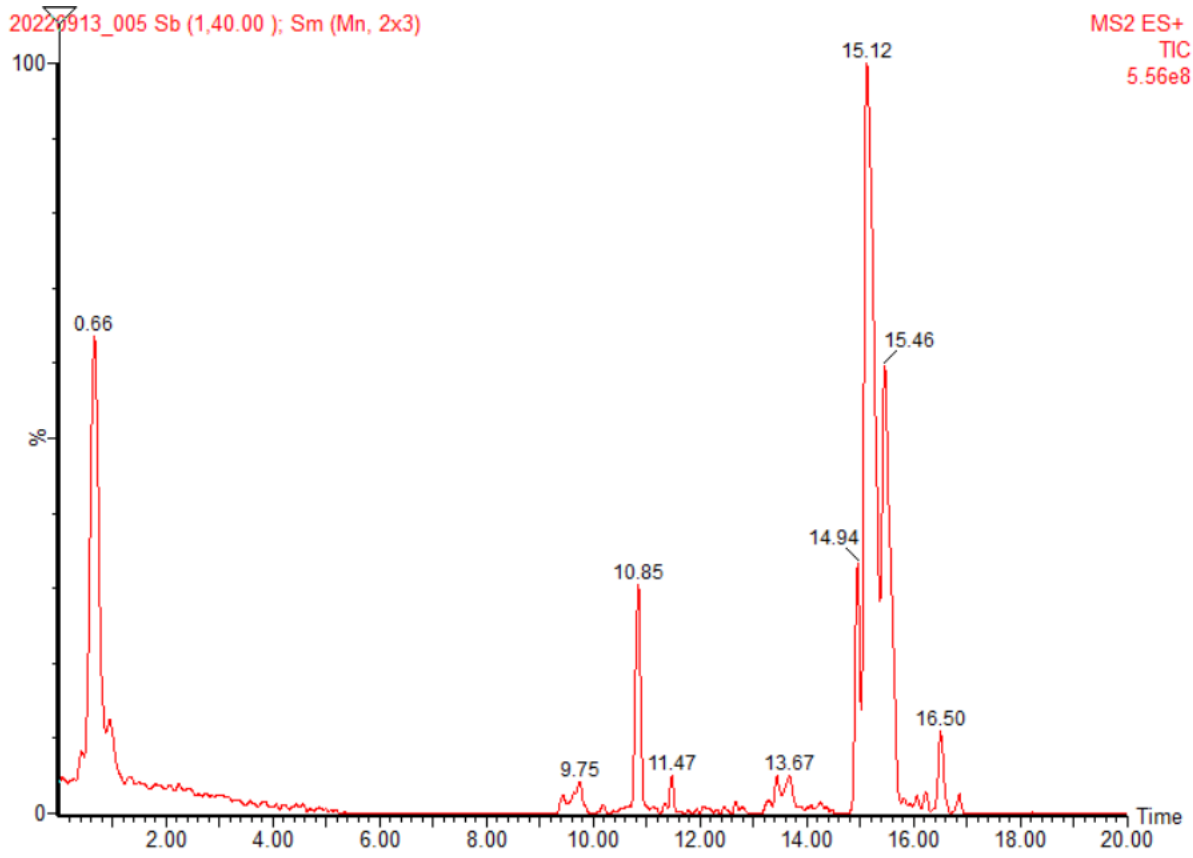

Figure S2. Peak chromatogram for a water:methanol (50:50) solution of mucilage extracted from yellow pitahaya fruit peels obtained by UPLC-QTOF-MS/MS.

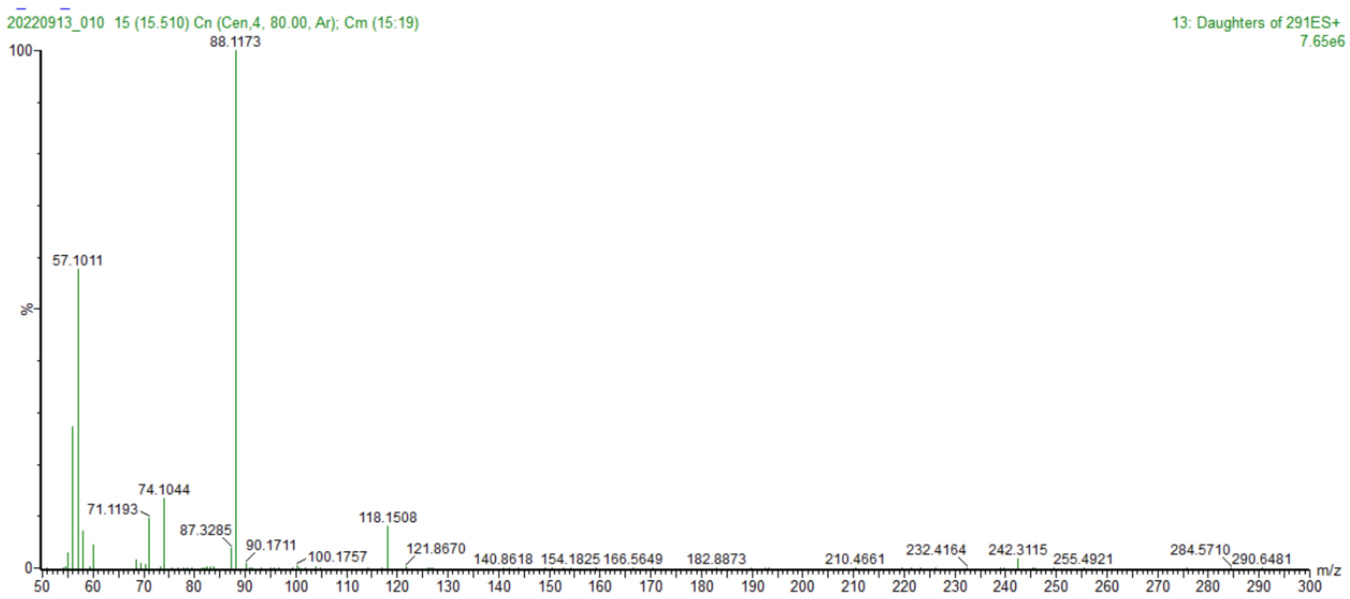

Figure S3. Positive ESI-MS spectrum of the component at r.t of 15.6 min of pitahaya fruit peel mucilage dissolved in water and measured at a collision offset voltage of 20 V.

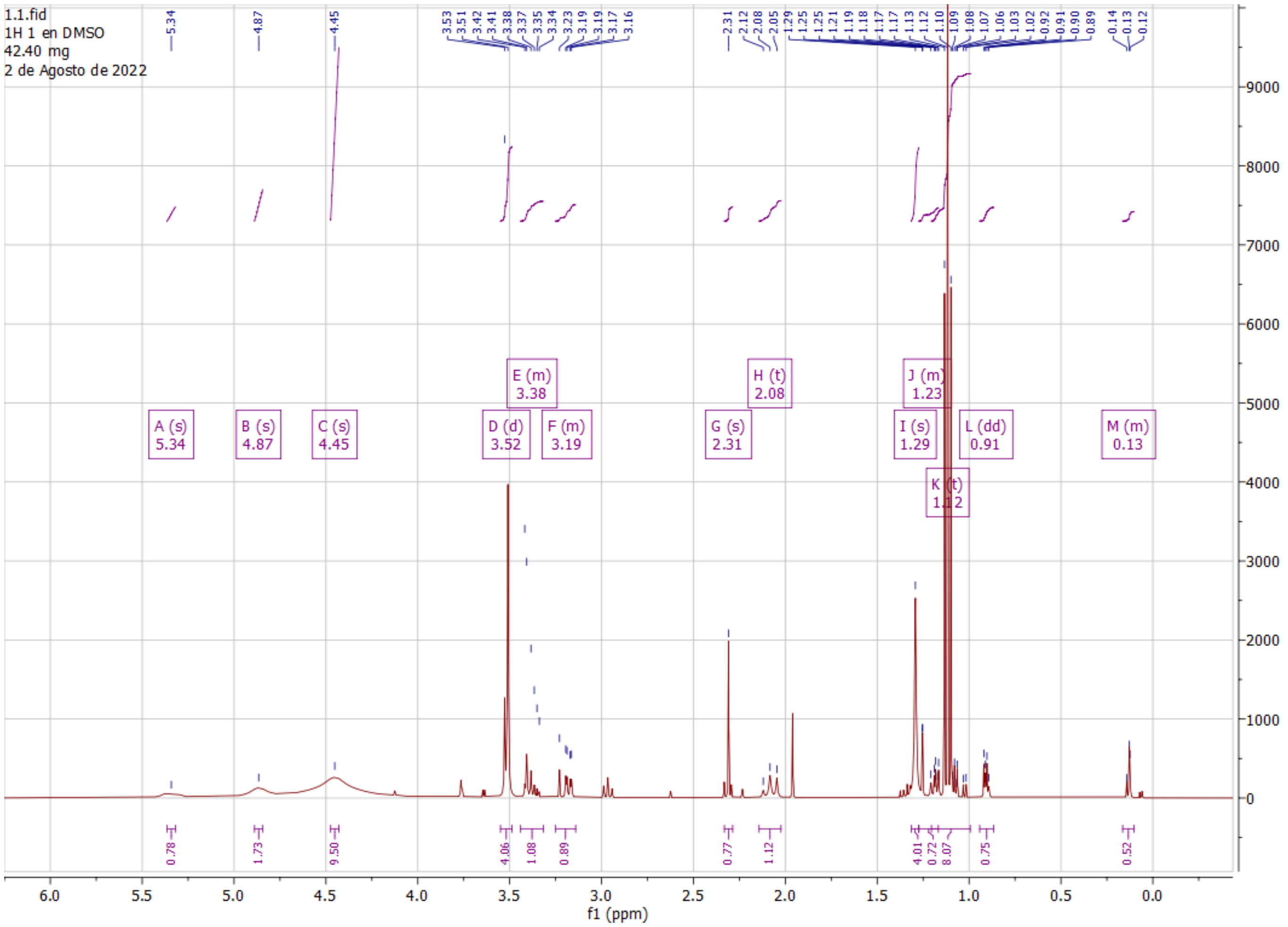

Figure S4. ^{1}H NMR spectrum (in DMSO-d6) of mucilage extracted from yellow pitahaya fruit peels.

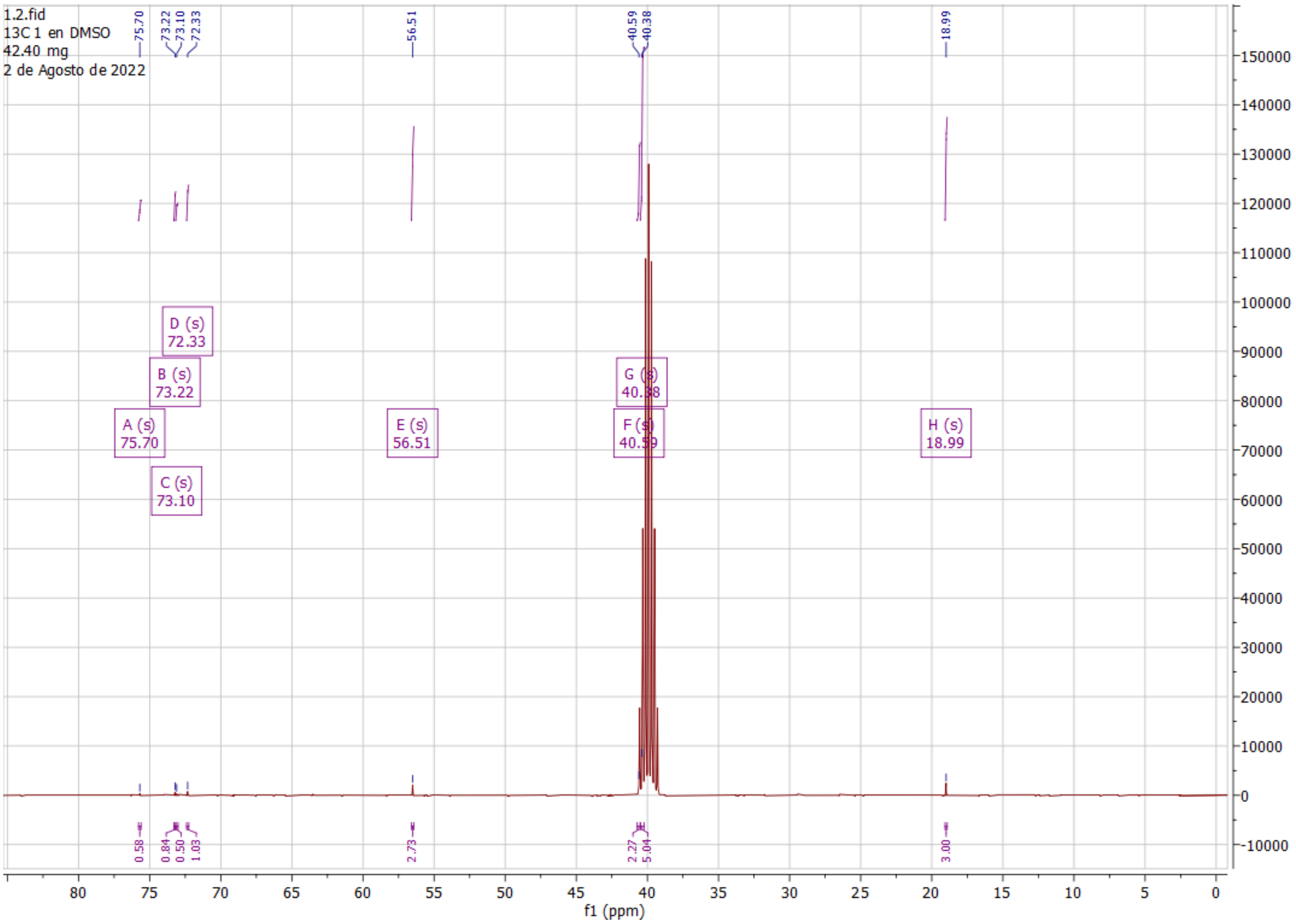

Figure S5. ^{13}C NMR spectrum (in DMSO-d6) of mucilage extracted from yellow pitahaya fruit peels.

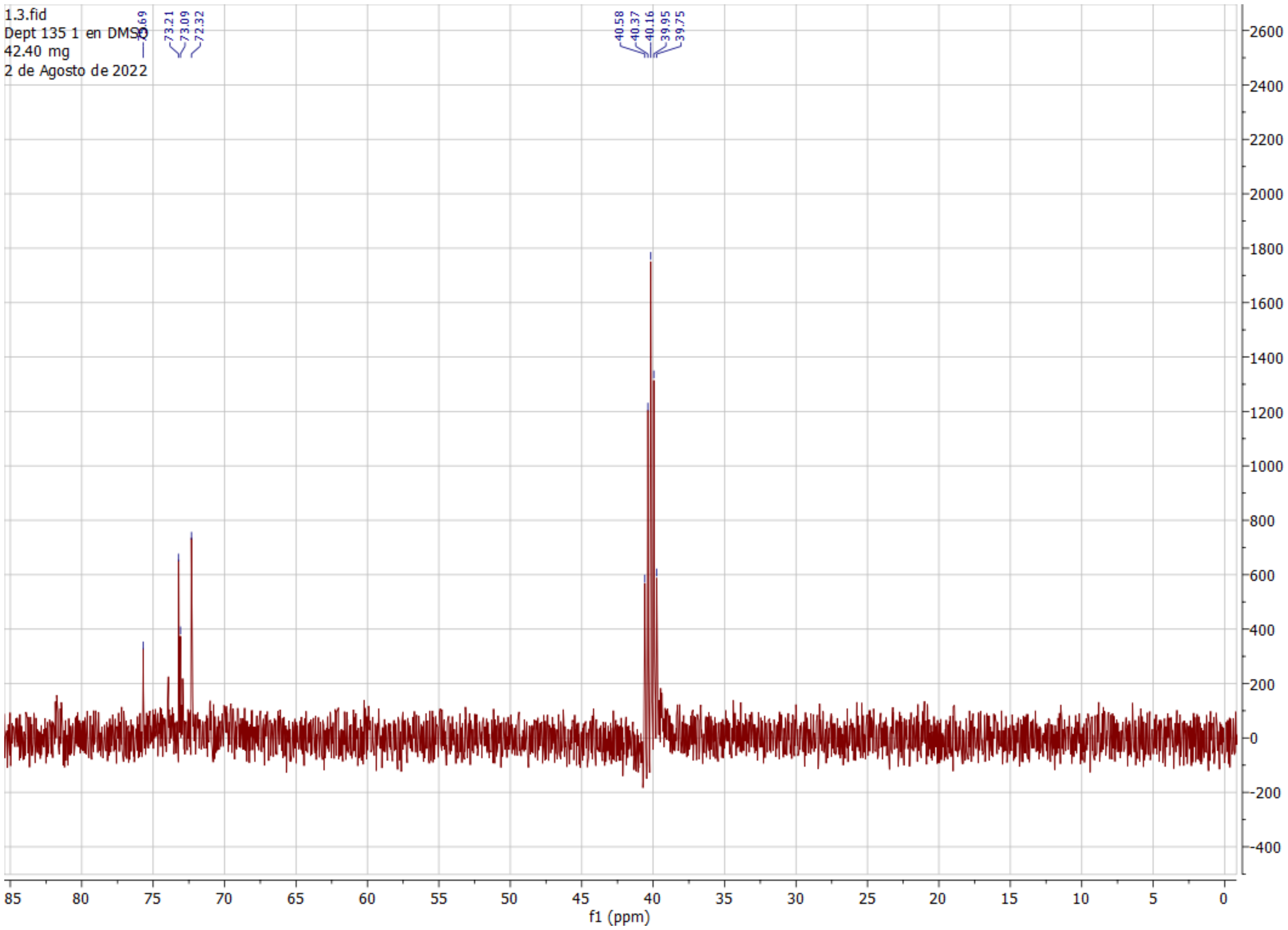

Figure S6. DEPT/13C NMR spectrum (in DMSO-d6) of mucilage extracted from yellow pitahaya fruit peels.

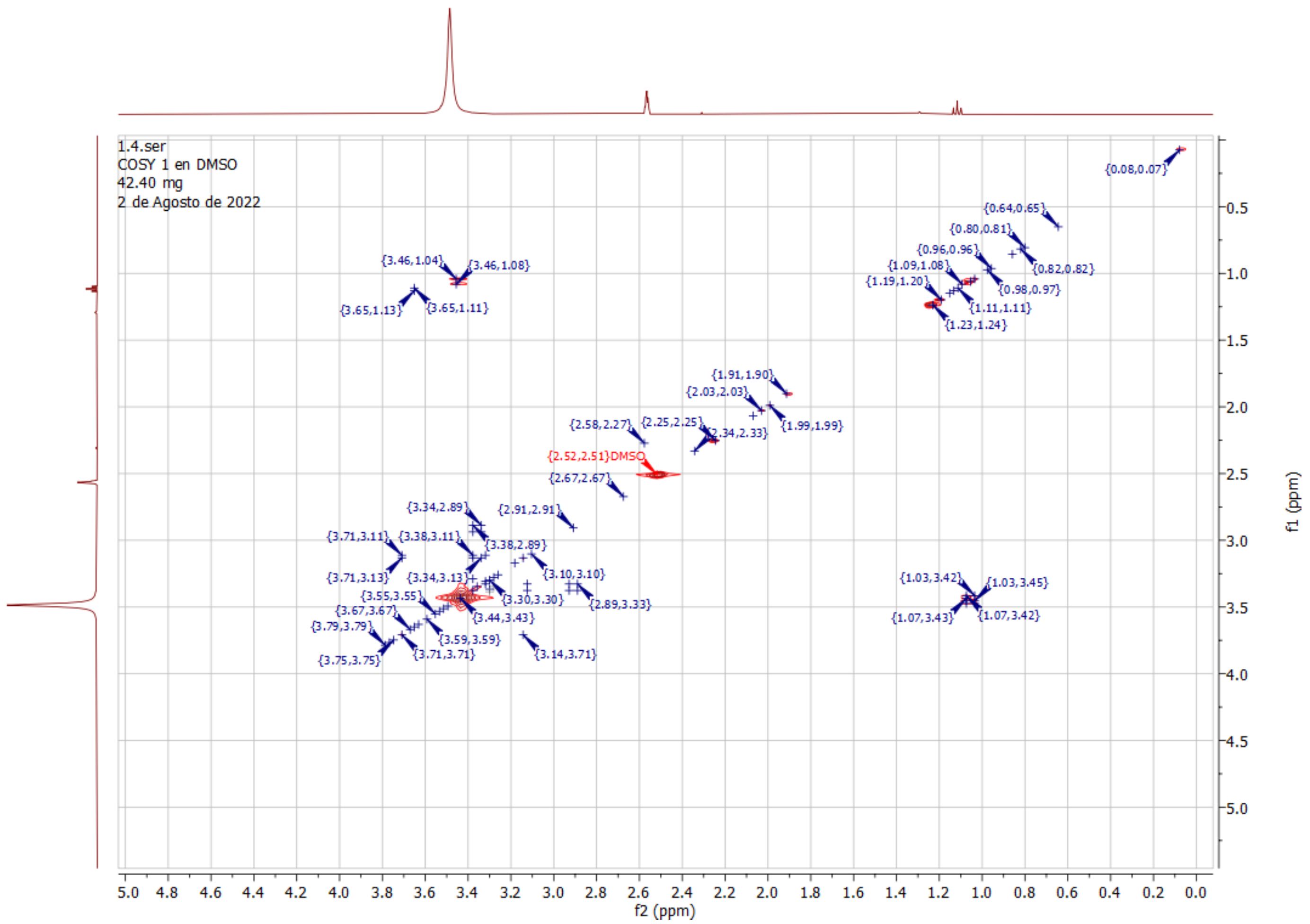

Figure S7. ^1H-^1H COSY NMR spectrum (in DMSO-d6) of mucilage extracted from yellow pitahaya fruit peels.

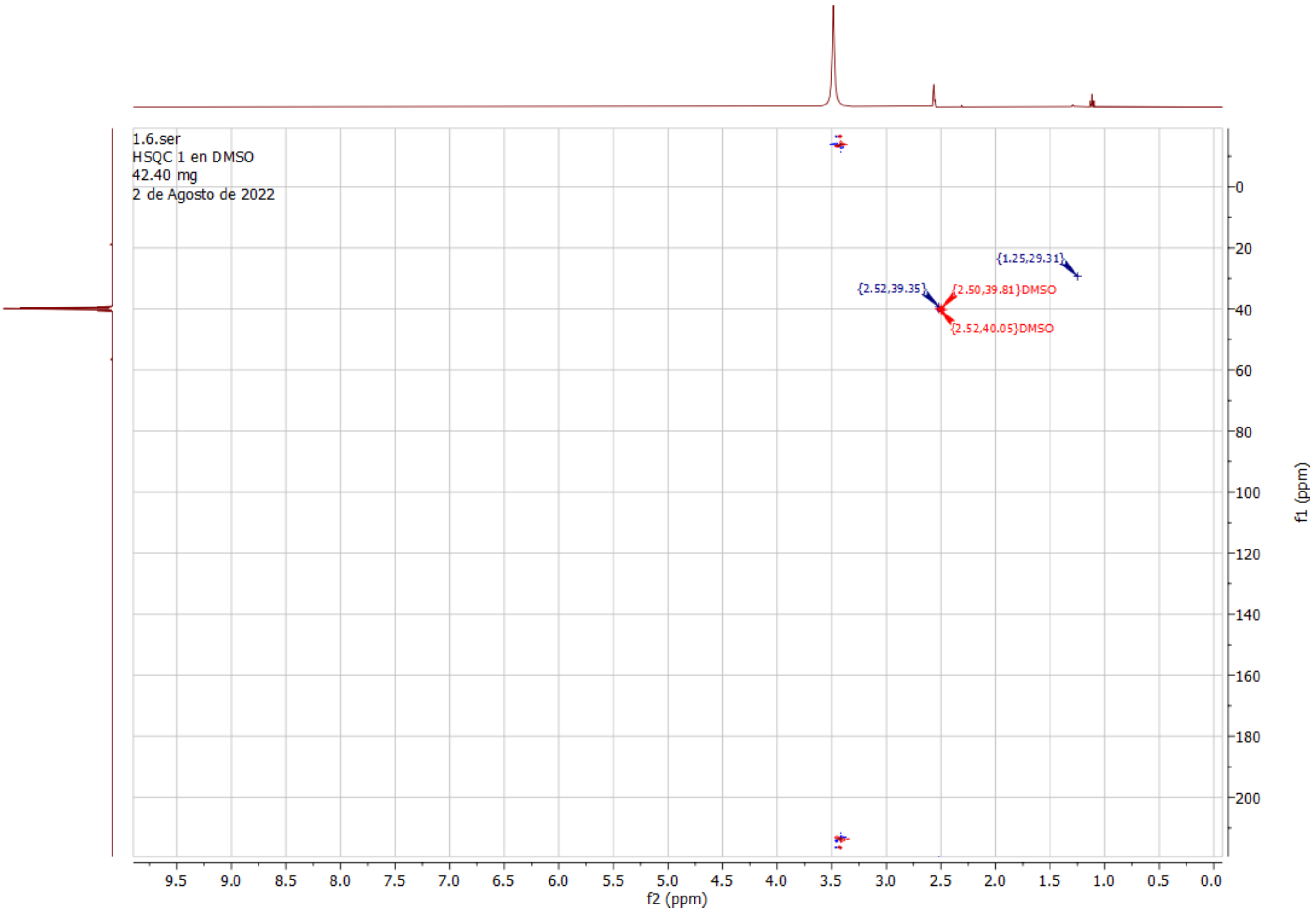

Figure S8. HSQC 2D-NMR spectrum (in DMSO-d6) of mucilage extracted from yellow pitahaya fruit peels.

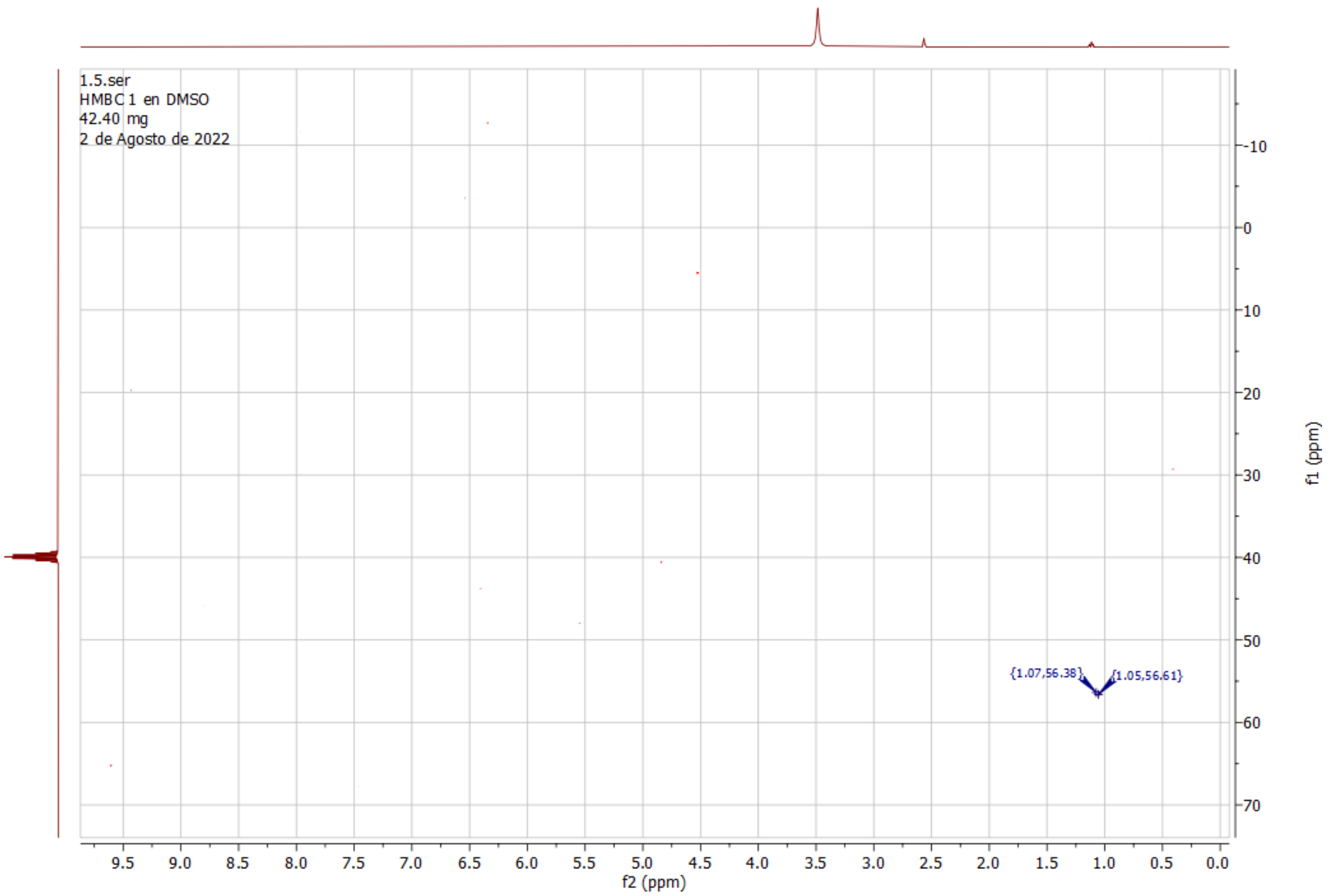

Figure S9. HMBC 2D-NMR spectrum (in DMSO-d6) of mucilage extracted from yellow pitahaya fruit peels.
